# Electrochemical Immunosensor for Human IgE Using Ferrocene Self-Assembled Monolayers Modified ITO Electrode

**DOI:** 10.3390/bios10040038

**Published:** 2020-04-14

**Authors:** Myungsang Park, Yesol Song, Ki Jun Kim, Seung Jun Oh, Jun Ki Ahn, Hun Park, Hang-Beum Shin, Seong Jung Kwon

**Affiliations:** 1 Department of Chemistry, Konkuk University, 120 Neungdong-ro, Gwangjin-gu, Seoul 05029, Korea; redeyes0011@konkuk.ac.kr (M.P.); sys9913@konkuk.ac.kr (Y.S.); kim5732@konkuk.ac.kr (K.J.K.); 2 Pioneering Research Center, LG Chem. Ltd., 30 Magokjungang 10-ro, Gangseo-gu, Seoul 07796, Korea; rilke@lgchem.com (S.J.O.); everjkahn@lgchem.com (J.K.A.); nagisa@lgchem.com (H.P.); 3 Human Convergence Technology Group, Korea Institute of Industrial Technology (KITECH), 143 Hanggaul-ro, Sangnok-gu, Ansan 15588, Korea

**Keywords:** human immunoglobulin E, indium tin oxide (ITO), electrocatalytic reaction, ferrocene, electrochemical impedance spectroscopy

## Abstract

The immunoglobulin E (IgE) level in serum is an important factor in the examination of allergy. Ferrocene (Fc)-modified self-assembled monolayers (SAMs) were placed on an indium tin oxide (ITO) electrode as a sensing layer for the detection of human IgE. The Fc moiety in the SAMs facilitated the electron transfer through the organic SAMs layer and electrocatalytic signal amplification. The electrochemical measurement was accomplished after the sandwich type immobilization of the receptor antibody, target human IgE, and enzyme conjugated secondary antibody. The enzyme product, *p*-aminophenol, was quantitatively analyzed by redox cycling via Fc. In addition, the electrochemical impedance spectroscopy (EIS) was investigated for the detection of IgE. The limit of detection (LOD), limit of quantification (LOQ), and dynamic range of the electrochemical sensor were 3 IU/mL, 10 IU/mL, and from 10 IU/mL to 100 IU/mL, respectively.

## 1. Introduction

Allergy (or allergic reactions) is a major cause of various allergic diseases, such as atopic dermatitis, allergic asthma, allergic rhinitis, urticaria, and food/animal/insect/plant allergies and has a significant effect on a person’s health [1,2,3,4]. The immune system generates antibodies (or immunoglobulin) to allergens during the first contact, and any additional contact triggers a violent defensive reaction. Immunoglobulin E (IgE), one of the five isotypes of immunoglobulin (A, G, M, D, E), plays an integrated role in this hypersensitivity [1,5,6].

IgE is synthesized by plasma cells like other immunoglobulin and is typically the least abundant isotype in blood, usually less than 1 IU/mL (1 IU = 2.4 ng) [6]. However, if an allergic reaction occurs, the IgE concentration increases. Thus, the test for the presence of allergy in humans is based mainly on the detection of allergen-specific IgE in serum [7]. The total IgE serum levels, as well as the allergen-specific IgE levels, are widely reported as a marker of allergic diseases, and are also used to monitor various anti-allergic therapies [8,9,10]. It is deemed positive for allergies when the allergen-specific IgE for a particular allergen is above a level of 0.35 IU/mL, or when the total IgE serum level is above ~100 IU/mL [1,6].

The majority of allergy tests nowadays are based on the antibody receptor (immunoassay). Common methods include radioimmunoassay [11], enzyme-linked immunoassay [12], and chemiluminescence immunoassay [13]. A wide range of other optical, spectroscopical, or electrochemical detection methodologies such as fluorescence microarray technology [14], matrix-assisted laser desorption ionization [15], atomic force microscopy [16], and quartz crystal microbalance [17], have been developed. Unlike other methods, electrochemical immunosensors have an advantage in terms of cost and miniaturization [18,19]. Some electrochemical IgE sensors presented a good performance with a low limit of detection (LOD) of ~1.5 IU/mL [20] or ~0.1 IU/mL [21].

In this paper, we investigated the electrochemical immunosensing of human IgE using cyclic voltammetry (CV) and electrochemical impedance spectroscopy (EIS). Even though Indium tin oxide (ITO), one of the most widely utilized conductive oxide thin films, presents an advantage in spectroelectrochemistry due to its transparent property, the ITO electrode is a still promising material for developing commercial electrochemical biosensors instead of noble metal electrodes such as Au electrodes, owing to its low electrical resistivity, low background current, wide potential window, applicability to self-assembled monolayers (SAMs), and cheaper price than Au electrodes [22]. Therefore, in this work, the ITO coated glass electrode was employed as a working electrode for both electrochemical detection methods instead of the Au electrode.

For the cyclic voltammetric detection, the electron transfer mediator Fc-modified SAMs were constructed on the ITO electrode. Next, streptavidin, biotin conjugated receptor antibodies, target human IgE, and alkaline phosphatase (ALP) conjugated secondary antibodies were immobilized in turn. Consequently, the electrochemical signal amplification by enzymatic reaction and redox cycling via Fc was successfully observed. 

Not only the CV, but also the EIS, was applied to investigate the IgE concentration. The EIS is a label-free and convenient tools for monitoring the charge transfer processes of immunosensors [23,24]. Therefore, the Nyquist plots were obtained and analyzed after the step of target IgE incubation without the binding of the secondary antibody.

## 2. Materials and Methods

### 2.1. Chemicals

(3-Aminopropyl)triethoxysilane (APTES), ferrocenecarboxaldehyde, sodium borohydride (NaBH_4_), streptavidin, 4-aminophenol (*p*-AP), 4-aminophenyl phosphate monosodium salt hydrate (*p*-APP), thrombin for human plasma, ferri/ferrocyanide, bovine serum albumin (BSA), human IgG, hemoglobin, horse serum, and all buffer salts were purchased from Sigma Aldrich (St. Louis, MO, USA), unless otherwise stated. NH_4_OH (30% diluted) and H_2_O_2_ (35% diluted) were obtained from Samchun (Seoul, Korea). Immunoglobulin E (IgE) human serum (Target IgE) was obtained from the National Institute of Biological Standards (NIBSC, Potters Bar, UK). Biotin-conjugated rabbit anti-human IgE (heavy chain) antibody (receptor antibody), alkaline phosphatase (ALP)-conjugated goat (or mouse) anti-human IgE antibody (secondary antibody), and goat immunoglobulin G (IgG), mouse IgG, and ALP-conjugated streptavidin were obtained from Thermo Fischer Scientific (Waltham, MA, USA). All chemicals were used as received. Ultrapure water (>18 MΩ, Millipore, Darmstadt, Germany) was used in all experiments.

### 2.2. Preparation of ITO Electrode and an Electrochemical Sensing Layer

ITO was obtained from Samsung Corning (Daegu, Republic of Korea). A cut of the ITO electrode was cleaned with ethanol and dried with N_2_ gas. To activate the hydroxyl group on the surface, the electrode was incubated in a solution [H_2_O:NH_4_OH:H_2_O_2_ = (v/v/v) 5:1:1] at 70 °C for 2 h, rinsed with distilled water, and dried with N_2_ gas [25].

For the formation of APTES SAMs, the cleaned electrode was incubated in a diluted APTES solution (APTES:ethanol = (v/v) 1:50) for 2 h, and washed with pure ethanol [26,27]. Next, the APTES SAMs-modified ITO electrodes were incubated in an ethanol solution containing 1 mM ferrocenecarboxaldehyde for 2 h. Here, the amine terminal of APTES reacted with the aldehyde group in ferrocenecarboxyaldehyde through the amine-aldehyde reaction, forming an imine. To make a stable bond, the imine, a carbon-to-nitrogen double bond, was reduced to the carbon-to-nitrogen single bond by incubating the electrode in a reducing agent solution (1 mg/mL of NaBH_4_) for 1 h. Then, the Fc-modified SAMs was obtained on the ITO electrode [28].

**Scheme 1:**
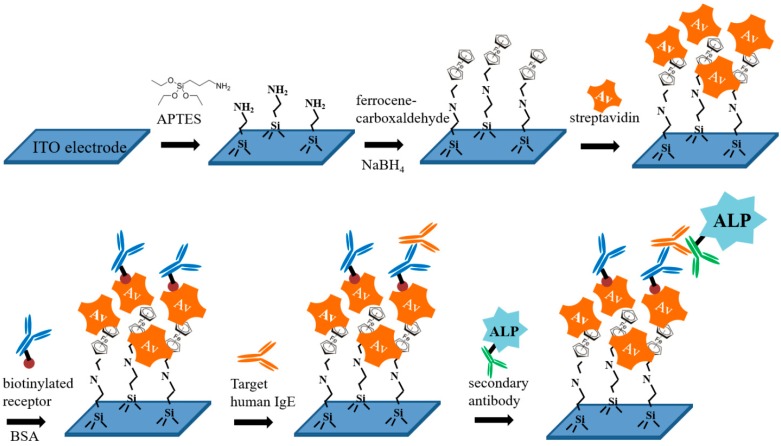
Schematic illustration of the formation of SAMs and the immobilization of antibodies on the ITO electrode.

The SAMs modified electrode was incubated in phosphate buffered saline (PBS) (1×, pH 7.4) solution containing 10 μg/mL of streptavidin for the immobilization of the receptor antibody. The streptavidin is addressed on the electrode surface through non-specific binding. After rinsing with PBS (1×, pH 7.4) buffer, the electrode was incubated in PBS (1×, pH 7.4) containing 10 μg/mL of biotin-conjugated receptor antibody for 1 h, after which the unreacted reagent was washed by PBS (1×, pH 7.4). The electrode was incubated in a PBS (1×, pH 7.4) containing 0.1% (*w*/*w*) BSA solution to prevent the non-specific binding of proteins for 0.5 h [29]. For the recognition with the target human IgE, the receptor-conjugated immunosensor was immersed in a PBS (1×, pH 7.4) containing various concentrations of target human IgE from 0 IU/mL to 300 IU/mL (1 IU = 2.4 ng), for 1 h. Next, the immunosensor was immersed in PBS (1×, pH 7.4) solution diluted at 10 μg/mL of ALP-conjugated secondary antibody for 1 h, and was rinsed with PBS (1×, pH 7.4). The whole process is shown in the Scheme 1.

For the control experiments, goat IgG (100 μg/mL), mouse IgG (100 μg/mL), thrombin (100 μg/mL), human IgG (1 mg/mL), and hemoglobin (5 mg/mL) were used instead of target IgE. Target IgE and the other reference materials were prepared using PBS (1×, pH 7.4) buffer.

### 2.3. Electrochemical Cell and Measurements

The electrochemical experiments, both CV and EIS, were performed by using a CHI 750 potentiostat (CH Instruments, Austin, TX, USA) with three electrode cells placed in a Faraday cage. The electrochemical cell consisted of a prepared ITO working electrode, a Pt wire counter electrode, and an Ag/AgCl (1 M KCl) reference electrode. All the potentials are reported vs. Ag/AgCl when not indicated differently.

CV was performed in an electrolyte solution consisting of 50 mM tris(hydroxymethyl)aminomethane (Tris), 10 mM KCl, and 1 g/L MgCl_2_ (pH 9.0) with/without 1 mM *p*-AP or 1 mM *p*-APP. The solutions of *p*-AP or *p*-APP were prepared daily before use. All the electrochemical measurements were obtained after having 10 min of quiet time for the enzymatic reaction.

EIS was performed using ferri/ferrocyanide (2.5/2.5 mM) as a redox probe in 1X PBS (pH = 7.4). A potential of 0.18 V was applied with a perturbation amplitude of 5 mV between frequencies of 1 MHz and 0.01 Hz. Although the Fc moiety was placed on the surface of the biosensor electrode, it was difficult to observe the apparent signal changes caused by the Fc reporting (the data is not shown here). This is because the surface concentration of Fc was too small, and the internal Fc was not sensitive to the externally attached target IgE. Therefore, the redox probe, ferri/ferrocyanide, was added into the solution for the EIS measurement.

## 3. Results and Discussion

### 3.1. Preparation of Fc-Modified SAMs on the ITO Electrode

In the electrochemical immunosensor, the use of self-assembled monolayers (SAMs) can provide a reproducible and robust path to functionalize electrode surfaces. Various organic molecules containing anchor groups such as thiols, amines, or silanes are used for the formation of SAMs depending on the electrode materials [30,31]. The silanes and phosphate anchor groups are available for the SAMs formation on the ITO electrode [25,26,32].

Generally, in electrochemical biosensors, because the additional organic layers of SAMs and biomolecules make the electron transfer difficult, redox-mediating functional groups that enable electron transfer between electroactive species and the SAMs-modified electrodes are required for the successive electrochemical detection. Therefore, the redox mediator-introduced SAMs can be an excellent platform for electrochemical biosensors.

In this research, we introduced the electron transfer mediator, Fc, as a functionality in the APTES SAMs-modified ITO electrode. In previous studies [33,34,35], the Fc-modified SAMs were constructed on the Au electrode by a complicated method: for example, using a specially designed Fc-derivative, such as an Fc-tethered dendrimer or Fc-functionalized thiols. Therefore, less reproducible mixed SAMs using multiple thiols and additional immobilization steps using a crosslinker were required for the introduction of the Fc moiety in the electrochemical sensing layer. However, in this work, we introduced the Fc moiety directly on the amine-SAMs by an aldehyde-amine reaction, which gave a simpler and more reproducible pathway for making an Fc layer than the previous methods did.

Then, the redox-active Fc SAMs-modified electrode was investigated as an immunosensor for the detection of human IgE. The combination of ALP and *p*-AP is employed as an enzyme label and a signal-reporting molecule for the electrochemical measurement [36]. The Fc moiety in the mixed SAMs shows an outstanding redox mediation for the oxidation of ALP-generated *p*-AP. Here, the electroactive species, *p*-AP, is generated by the enzyme label, ALP, from its substrate, *p*-APP.

**Figure 1:**
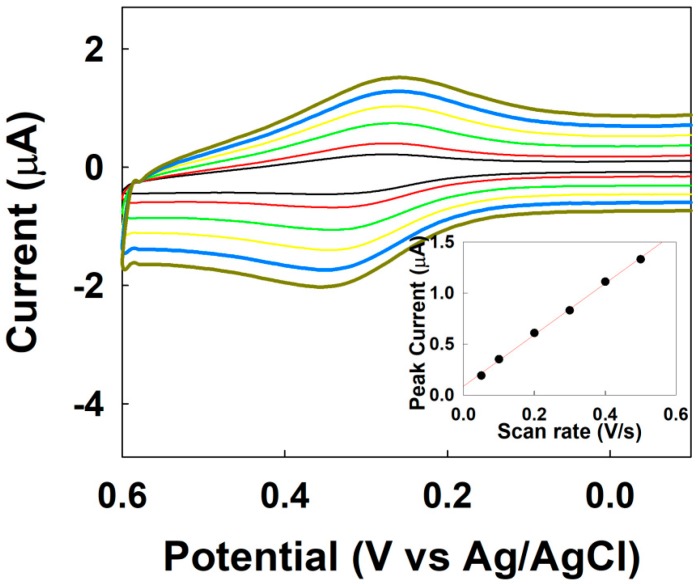
Cyclic voltammograms of the Fc SAM-modified ITO electrode with various scan rates in the electrolyte solution. Inset is a plot of the oxidation peak current as a function of the scan rate.

First, the redox reaction of the Fc moiety on the electrode surface was investigated to characterize the formation of the Fc-functionalized SAM onto the ITO electrodes. As shown in Figure 1, the cyclic voltammograms of the surface-bound Fc’s redox reaction were obtained.

The full width at half-maximums (FWHM) was ~160 mV, relatively larger than the ideal value for reversible responses, 90.6 mV [37]. The oxidation peak currents were proportional to the scan rate. However, the slope of the regression line (or proportional constant) was ~0.8, not exactly 1. Considering the numbers and shape of the CV, the Fc-functionalized SAMs seem to have somewhat diffusional properties for the Fc. The relative looser packing of SAMs on the ITO electrode than for the Au electrode makes the Fc more flexible, which may result in a broad peak shape compared to the ideal bell shape obtained typically by surface-bound species. The surface coverage of the Fc was calculated from the coulometric charge by integrating the anodic/cathodic peak area into the CV. Assuming that all the Fc sites are electrochemically active, the calculated surface concentrations of Fc (±standard deviation), *Γ*_s_, were 4.1 (±1.0) × 10^−7^ mol/cm^2^.

### 3.2. Electrocatalytic Amplification via Redox Cycling

**Figure 2:**
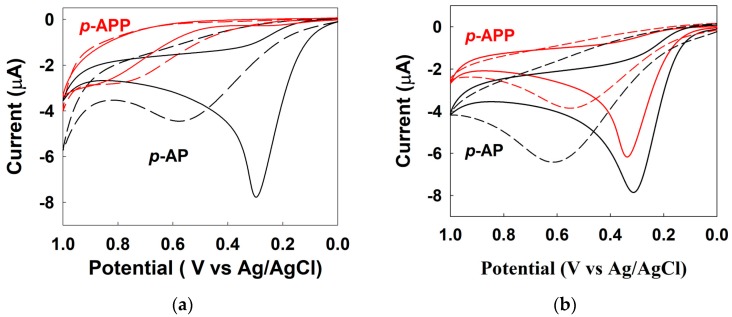
(**a**) Cyclic voltammograms of the SAM-modified ITO electrode with/without (solid/dashed line) the Fc moiety in an electrolyte solution containing 1 mM *p*-AP (Black) or 1 mM *p*-APP (red). Scan rates were 10 mV/s. (**b**) Cyclic voltammograms of the SAM-modified ITO electrode with/without (solid/dashed line) the Fc moiety after incubating with ALP-conjugated streptavidin in an electrolyte solution containing 1 mM *p*-AP (**Black**) or 1 mM *p*-APP (**Red**). Scan rates were 50 mV/s.

As shown in Figure 2a (solid line), the Fc SAMs-modified ITO was investigated in an electrolyte solution containing *p*-AP or *p*-APP to characterize the electrocatalytic redox cycling performance of the Fc-functionalized SAMs layer. Even if the *p*-APP existed, the electrocatalytic reaction between *p*-APP and Fc did not appear. The background current of *p*-APP was obtained only at the potential region of 0.0 V to 0.5 V. However, in the presence of *p*-AP, the oxidation part of the current was amplified by the electrocatalytic redox-mediated oxidation of *p*-AP, even though the reduction part of the current stayed intact. The mechanism is as follows: the Fc moiety in the SAMs is oxidized to the ferrocenium ion (Fc^+^) by the electrochemical oxidation reaction. Then, the Fc^+^ is reduced back to Fc by the chemical redox reaction with *p*-AP. As discussed in a previous report, this can be explained by the following mechanism [37,38]:

(1) $$2\;Fc\;\to \;2Fc^{+}+2\;e^{-}$$

(2) $$2Fc^{+}+p–AP\;\;\;\to \;\;quinonimide\;\left(QI\right)+2\;Fc+2\;H^{+}$$

where quinonimide (QI) is a molecule of *p*-AP that has been oxidized by the loss of two electrons. This redox cycling would continue until all the *p*-AP is spent. As a result, an amplified oxidation current proportional to the concentration of *p*-AP was obtained. In the absence of the electron transfer mediator, Fc, the electrochemical oxidation of *p*-AP was obtained at a higher potential region (~0.6 V), as shown in Figure 2a (dashed line). Since a considerable oxidation of *p*-APP occurs at this potential region, it is difficult to distinguish the signal current by the *p*-AP from the background current by the reactant *p*-APP. Therefore, the electron transfer mediator, Fc, is required.

To investigate the electron transfer mediation of Fc with the enzymatic reaction, the Fc SAM-modified electrode was incubated in a PBS containing 10 μg/mL of ALP-conjugated streptavidin for 1 h. As shown in Figure 2b (solid line), the Fc SAM layer with the presence of ALP on the surface was examined. In the solution containing *p*-APP, the electrocatalytic amplified current appeared at the same potential region as in the case of *p*-AP, indicating that *p*-APP was converted to *p*-AP by the enzymatic reaction. Therefore, in the presence of both *p*-APP and ALP, as in the case of *p*-AP, the electrocatalytic amplified current of Fc occurred at a ~0.3 V potential region. Consequently, the electron transfer mediator, Fc, plays an important role in the reduction of the reaction overpotential and in the amplification of the catalytic current. This is the expected operation of the Fc SAMs layer in the biosensor.

### 3.3. Electrochemical Detection of Human IgE via CV

Recently, a direct introduction of avidin on an electrode surface using non-specific binding was used as a simple and effective immobilization for biotin-labeled (bio)molecules on ITO [39,40]. Therefore, in this work, the prepared Fc-functionalized SAM-modified ITO electrode was incubated in a buffer containing streptavidin, as shown in Scheme 1. Then, the electrode was incubated in the biotin-conjugated receptor antibody. The receptor antibody was introduced on the electrode surface via an avidin-biotin interaction. Next, the prepared sensing layer was incubated in various concentrations of target human IgE. After rinsing with PBS buffer, the electrode was incubated in the enzyme-labeled secondary antibody solution. Therefore, a sandwich-type immunosensor was established.

**Scheme 2:**
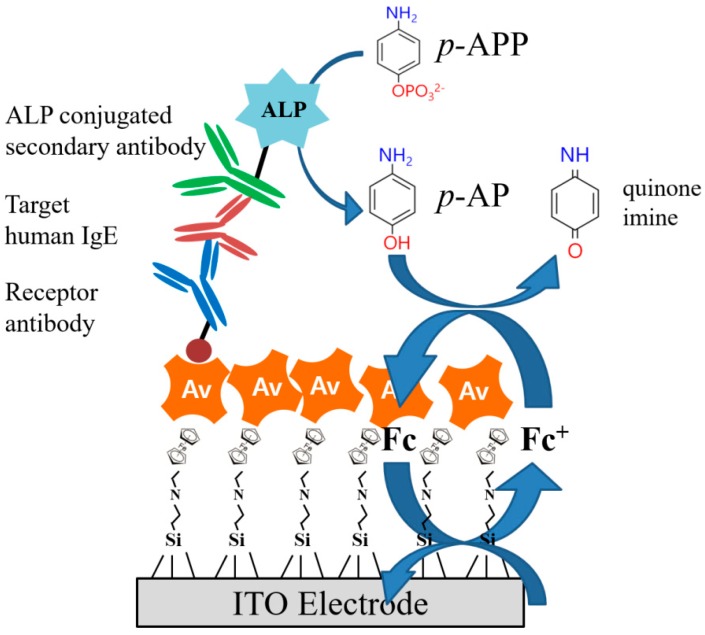
Schematic illustration of an electrochemical amplification of the immunosensor using redox-cycling.

For the electrochemical measurement, the prepared immunosensor was investigated by CV in the electrochemical buffer containing *p*-APP after 10 min of enzymatic reaction time. The electrochemistry of the sensing mechanism is depicted in Scheme 2. First, the *p*-APP was dephosphorylated to *p*-AP by the enzyme label, ALP, after which the *p*-AP was diffused to the nearby electrode surface by a concentration gradient. At the electrode surface, the Fc moiety of the SAMs was electrochemically oxidized to Fc^+^ by the electrode, and it reacted with the nearby *p*-AP. The redox cycling, as mentioned above, will be maintained until all the *p*-AP is consumed. Consequently, the amplified electrocatalytic current is proportional to the concentration of *p*-AP.

**Figure 3:**
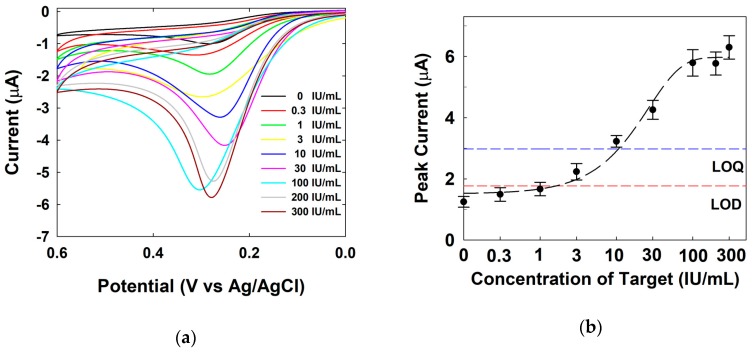
(**a**) Cyclic voltammogram of a sandwich-type electrochemical immunosensor for human IgE at various concentrations of target human IgE from 0 IU/ml to 100 IU/ml. Cyclic voltammograms were obtained after 10 min of enzymatic reaction time in buffer containing 1 mM *p*-APP at a scan rate of 10 mV/s. (**b**) Calibration curve for the oxidation peak current depending on the target concentration. The red dashed line represents the LOD obtained by the mean current at the zero concentration of the target IgE plus three times the standard deviation (SD). The blue dashed line represents the LOQ obtained by the mean current at the zero concentration of the target IgE plus ten times the SD. Each concentration experiment was performed at least five times. For the zero concentration (blank sample), the experiments were performed ten times.

The amplified electrocatalytic current was obtained in relation to the concentration of target human IgE, as shown in Figure 3. The obtained peak current was plotted as a function of the target IgE concentrations to determine the analytical characteristics, such as the limit of detection (LOD), limit of quantification (LOQ), and dynamic range. The LOD and LOQ were 3 IU/mL and 10 IU/mL, respectively. The dynamic range was from 10 to ~100 IU/mL. The calculated percent relative standard deviations (%RSD) were 14%, 15%, 13%, 12%, 6%, 7%, 7%, 7%, and 6% for the various target concentrations from 0 to 300 IU/mL, respectively. In clinical medicine, if the concentration of antigen-specific IgE is higher than 0.35 IU/mL or the concentration of total IgE is higher than 100 IU/mL, it is determined that there are allergic symptoms. Therefore, the LOQ of our immunosensor satisfied the requirement for the detection of the total IgE level [1,6].

### 3.4. Selectivity Test for the Electrochemical Sensing 

**Figure 4:**
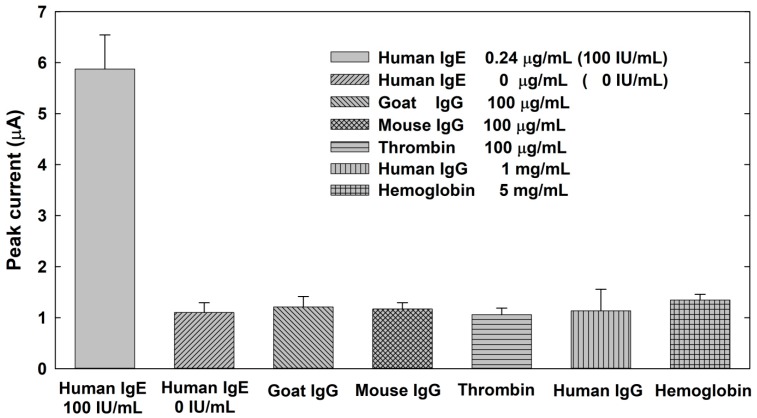
Selectivity tests using different targets: goat IgG, mouse IgG, thrombin, human IgG, hemoglobin, 0 IU/mL of target IgE, and 100 IU/mL of target IgE. Each experiment was performed at least three times.

In order to confirm the selectivity of the immunosensor for the target IgE, we assembled electrochemical sensors detecting other biomolecule targets such as goat IgG, mouse IgG, thrombin, human IgG, and hemoglobin. Then, when each sensor was measured in a solution containing 1 mM *p*-APP, the oxidation current was similar to the background current level of the target IgE case, as shown in Figure 4. In particular, the sensor showed good selectivity with the human IgG and hemoglobin, which are common in human blood serum. It was confirmed that the electrochemical sensor fabricated in this study had selectivity for the target IgE.

### 3.5. Stability Test for the Electrochemical Sensing

**Figure 5:**
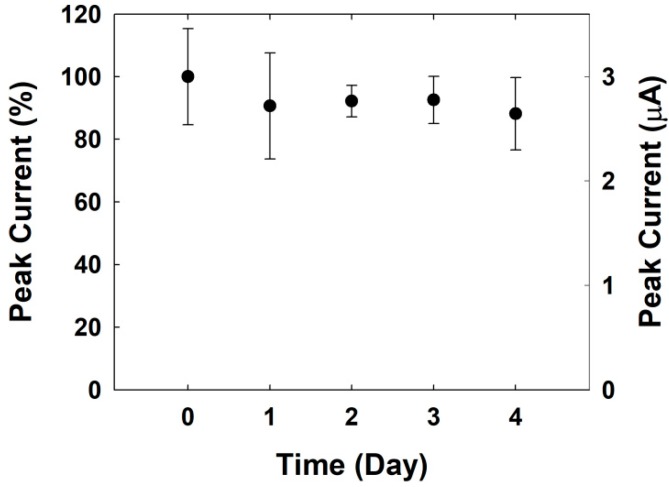
Current signal change as a function of time. The electrodes were prepared on the same day, but the bio-assay and electrochemical measurement were performed a few days later. The target IgE concentration was 10 IU/mL. Each experiment was performed at least three times.

The shelf life of the biosensor was investigated. The Fc-SAM-modified ITO electrodes were prepared on the same day. A few days later, the Fc-SAM-modified ITO electrodes were constructed by bio-reagents, and the electrochemical measurement were carried out. The biosensor layer consists of the organic SAM-modified inorganic material electrode; therefore, the electrode surface is expected to be very stable even for long-term storage. As shown in Figure 5, the peak current maintains its level. The magnitude of the current almost remains at the initial value over a few days when considering the error bar (SD).

### 3.6. Electrochemical Detection of Human IgE via EIS

The redox-marker and label are usually required for the electrochemical measurement. The requirement of this specific marker and label makes the electrochemical system more complicated than the other methods, resulting in the electrochemical sensor being uncompetitive. The electrochemical biosensor has advantages in terms of price and miniaturization, and therefore there have been many studies conducted to solve this marker and label problem. The EIS, a label-free electrochemical method, is one of the alternatives [23,24].

**Figure 6:**
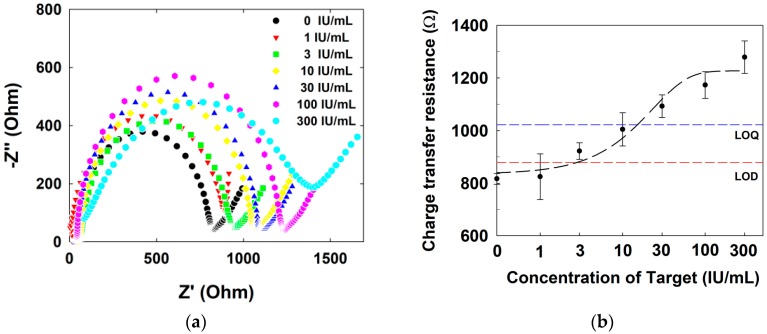
(**a**) Nyquist plot of the electrochemical immunosensor for human IgE at various concentrations of the target IgE from 0 IU/mL to 300 IU/mL. Plots were obtained in a 1× PBS containing ferri/ferrocyanide (2.5/2.5 mM) as a redox probe. (**b**) Calibration curve for the charge transfer resistance depending on the target IgE concentration. The red and blue dashed lines represent the LOD and LOQ, respectively. Each concentration experiment was performed at least five times.

EIS was tried with the exact same sensor after target immobilization, without the secondary antibody incubation step. Depending on the amount of target IgE immobilized on the sensor surface, the thickness of the insulating layer by the biomolecule changed. The redox reaction of a redox probe, ferri/ferrocyanide, was perturbed by the insulating layer on the electrode surface, resulting in a change of charge transfer resistance. As shown in Figure 6, the charge transfer resistance of the immunosensor was investigated and plotted as a function of the concentration of target IgE. The calibration curve is also presented in Figure 6b. The LOD and LOQ were 3 IU/mL and 30 IU/mL, respectively. The dynamic range was from 30 to ~100 IU/mL. The calculated percent relative standard deviations (%RSD) were 2%, 11%, 3%, 6%, 4%, 4%, and 5% for the various target concentrations from 0 to 300 IU/mL, respectively.

The EIS method is time- and cost-effective for the detection of IgE because it is label-free. However, the results were less reproducible than the CV measurements because all kinds of non-specific binding can affect the charge transfer resistance in EIS methods. To improve the selectivity and the sensitivity of EIS measurement, a probe molecule with a high binding affinity should be used, and a special pre-treatment or method to reduce the non-specific binding should be introduced.

We also investigated both electrochemical methods in a condition using a serum matrix. Human serum and horse serum were used as a matrix for the dilution of target human IgE. However, the noise background signals at 0 IU/mL of the target concentration were, in both serums, considerably high. Consequently, the analytical properties, such as the LOD or dynamic range, will be poor in serum media (the data is not shown here). Therefore, pre-treatments such as filtration or separation are required for application in real serum.

Interestingly, the detection limit of the EIS method was also ~3 IU/mL, close to the CV measurement results, despite the absence of an electrocatalytic amplification process in the EIS measurements. Because the same receptor antibody and target IgE were used, the observed similar analytical characteristics may indicate that the binding constant between the receptor antibody and the target IgE is the dominant variable for determining the selectivity of the immunosensor, rather than other factors such as the signal amplification. The sensing ability of the immunosensor actually varied greatly depending on the type of receptor antibody. Further studies may be needed on the competitive effects between the receptor’s binding affinity and the signal amplification scheme in relation to the sensitivity of the biosensor.

## 4. Conclusions

We introduced the electron transfer mediator-functionalized layer on the ITO electrode for the electrochemical immunosensing of human IgE. The electron-transfer mediator, Fc, was directly introduced on the ITO electrode by an aldehyde-amine reaction. The formation of Fc SAMs-modified ITO electrodes is easier and cheaper than that of a similar Fc modification on Au electrodes using mixed SAMs.

The electron-transfer mediation of the Fc SAMs-modified ITO electrode was measured by using a *p*-AP oxidation reaction. The electrocatalytic current amplification was achieved firstly with enzymatic production and secondly with redox cycling by Fc. The LOQ of the immunosensor was 10 IU/mL of the target IgE. This is good enough to determine the allergy based on the total IgE level. In addition to this, a label-free EIS measurement was also applied, obtaining a similar level of sensitivity. Therefore, the electrochemical detection of human IgE was successfully obtained via both CV and EIS.
